# Oleuropein Induces AMPK-Dependent Autophagy in NAFLD Mice, Regardless of the Gender

**DOI:** 10.3390/ijms19123948

**Published:** 2018-12-08

**Authors:** Cristiana Porcu, Silvia Sideri, Maurizio Martini, Alessandra Cocomazzi, Andrea Galli, Giovanni Tarantino, Clara Balsano

**Affiliations:** 1 F. Balsano Foundation, Via Giovanni Battista Martini 6, 00198 Rome, Italy; cristiana.porcu@live.it; 2 MESVA Department, University of L’Aquila, Piazza S. Salvatore Tommasi 1, 67100 Coppito, L’Aquila, Italy and F. Balsano Foundation, Via Giovanni Battista Martini 6, 00198 Rome, Italy; silviasideri@gmail.com; 3 Fondazione Policlinico A. Gemelli, IRCCS, Università Cattolica del Sacro Cuore Rome, Italy; maurizio.martini@unicatt.it (M.M.); alessandra.cocomazzi@libero.it (A.C.); 4 Gastroenterology Unit, Department of Experimental and Clinical Biomedical Sciences “Mario Serio”, University of Florence, 50139 Florence, Italy; a.galli@dfc.unifi.it; 5 Department of Clinical Medicine and Surgery, Federico II University Medical School of Naples, 80131 Naples, Italy; tarantin@unina.it

**Keywords:** autophagy, AMPK, mTOR, oleuropein, phenolic compound, nutraceutical, Mediterranean diet, olive oil, liver steatosis, NAFLD

## Abstract

Oleuropein (Ole) is one of the most plentiful phenolic compounds with antioxidant, anti-inflammatory, anti-atherogenic, hypoglycemic and hypolipidemic effects. The aim of our study was to establish whether the positive Ole-related effects on liver steatosis could be associated with autophagy. Female and male C57BL/6J mice were fed normal diet (ND) or high-fat diet (HFD) for eight weeks, and Ole was added or not for the following eight weeks. The autophagy-related proteins Akt, mTOR, AMPK, ULK1, Beclin-1, LC3B and p62/Sqstm1 were analyzed. Interestingly, Ole induced a different regulation of the Akt/mTOR pathway in female compared to male mice, but was able to activate the autophagic process in ND and HFD mice through AMPK-dependent phosphorylation of ULK1 at Ser555, regardless of the gender. Our work reveals the ability of Ole to induce, in liver of ND and HFD mice, autophagy independently by gender-specific mTOR activation. We highlight Ole as a novel therapeutic approach to counteract unhealthy diet-related liver steatosis by targeting autophagy.

## 1. Introduction

Non-alcoholic fatty liver disease (NAFLD) is a major form of chronic liver disease and affects about 25% of the world population. NAFLD is considered the hepatic manifestation of metabolic syndrome, highly associated with obesity and insulin resistance [1]. NAFLD and obesity share common clinical aspects and similar physiopathologic mechanisms. NAFLD, as well as NASH (non-alcoholic steatohepatitis), are highly prevalent in all continents, but epidemiology and demographic characteristics vary worldwide, with about 32% of prevalence in South America and the Middle East, 27% in Asia and 23% in the USA and Europe [2].

NAFLD/NASH, in the next future, will emerge as the leading cause of end-stage liver disease, thus the development of effective therapies warrants special attention. In this context, plant polyphenols, such as oleuropein (Ole), which are often part of the so-called Mediterranean diet and have important healthy protective effects [3,4], might be a remarkable resource to be taken into account.

It is well known that apoptosis contributes to the pathogenesis of NASH, and apoptosis-related caspase family molecules play important roles in the progression of liver disease [5], whereas, in the steatotic liver, autophagy has been described to be less effective [6]. However, it is still unclear how autophagy is regulated during unhealthy diet and its crosstalk with cell death [7,8,9,10]. In fact, although autophagy surely plays a protective role under a physiologic condition by degrading damaged mitochondria and protein aggregates, the activation of autophagy for a long time, as happens under pathologic conditions, can lead to organ dysfunction by degrading essential proteins and organelles [11].

The word “autophagy” means self-eating and derives from the ancient Greek: to eat (“phagy”) self (“auto”). Autophagy occurs in multiple different cell types and is activated either under cellular starvation or under nutrient overload. Autophagy is a safeguard cellular mechanism involved in intracellular protein degradation and characterized by autophagosome formation [12]. Besides its important role through bulk degradation in supplying nutrients, including lipid overload, this system has the capacity to degrade certain proteins and organelles to maintain cellular homeostasis [13]. During the past decade, a great deal of knowledge of autophagy has been gained, showing that the function of autophagy is complex and involved in a lot of different processes, such as cellular differentiation, regulation of metabolism, aging and cellular defense [14,15,16,17,18,19].

Previous studies have highlighted that AMP-activated protein kinase (AMPK) is an autophagy inducer by Atg1/Unc-51-like-kinase1 (ULK1) phosphorylation (Ser 555) [20]. ULK1 is required for full autophagic induction and activates, in turn, Beclin-1, which has a central role in stimulating autophagy due to cellular stress. At the early stage of autophagy, Beclin-1 collaborates to form the autophagosome by creating the isolation membrane, a double-membrane structure that surrounds cytoplasmic material [21]. Efficient macro-autophagic responses have been associated with the activity of ATG3 and ATG7, which together with the ATG12–ATG5: ATG16L1 complex conjugates phosphatidylethanolamine to microtubule-associated protein 1 light chain 3 beta (LC3B) [22,23]. Protein light chain 3 (LC3) and p62/Sequestosome 1 (p62/Sqstm1) are linked to autophagosomal membranes and participate in cytoplasmic content degradation [22].

Ole (*Olea europaea L.)* is a non-toxic secoiridoid phenol present in leaves and fruits of olives. Ole represents up to 14% of the olive fruit’s dry weight, being one of the most abundant phenols in olives [24]. Recently, Park et al. [25] highlighted hepato-protective effects of Ole in a mouse model fed a high-fat diet (HFD) and characterized by the presence of liver steatosis.

Bioavailability and metabolism of Ole are heterogeneous and highly dependent on a number of factors, including gender. Ole displays important gender-related differences in membrane transport that have been reported in various organs of the body, including liver, kidney, intestine and brain. These sex-related differences in transport systems are definitely involved in the inter-individual variability of pharmacokinetics and pharmacodynamics [26]. Moreover, large inter-individual variations in absorption and metabolism of phenolic compounds have been reported, as a consequence of different enzymatic activity of the liver [27]. Indeed, the daily inter-individual variation, due to circadian rhythm, makes it even more difficult to develop reliable data on molecules such as Ole [28]. Thus, when studying this kind of compound, it is extremely important to carefully evaluate sex differences.

Ole down-regulates the expression of numerous genes involved in hepatic lipogenesis, oxidative stress, and pro-inflammatory response, however it has been never investigated if the ability of Ole in improving liver steatosis might be related to the activation of autophagy. 

To achieve this aim, we have examined autophagy response to Ole in NAFLD mice at 16-weeks, taking into account sex-dependent responses to HFD. The expression levels of autophagy-related proteins Akt, mTOR, AMPK, ULK1, Beclin-1, LC3B and p62/Sqstm1 were contextually studied.

## 2. Results

### 2.1. ND and HFD Liver Histology before and after Ole Treatment

We used C57BL/6J murine models that well mimic human metabolic syndromes, developing steatosis, metabolic and cardiovascular diseases [29].

**Figure 1:**
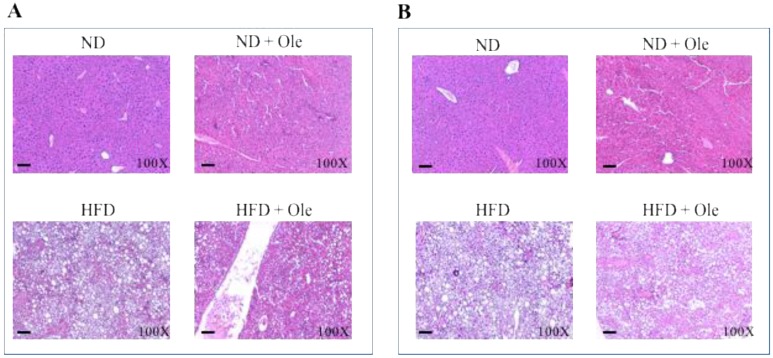
Effects of Ole treatment on liver histology in normal diet (ND) or high-fat diet (HFD) mice. Hematoxylin and eosin staining in liver sections from representative liver tissues of female (**A**) and male (**B**) mice fed ND or HFD in presence or absence of Ole. Original magnification × 100, scale bar = 80 μm.

First of all, we performed hematoxylin and eosin stain of liver tissues of ND and HFD female (Figure 1A) and male (Figure 1B) mice treated or not with 3% of Ole, administered by oral gavage. As expected, Ole was able to improve liver steatosis either in females or males (Figure 1A,B). 

However, HFD induced a prevalent aspect of micro-vacuolar liver steatosis in females (about 60% micro-vacuolar and 40% of macro-vacuolar liver steatosis, Figure 1A) and, conversely a prevalent aspect of macro-vacuolar liver steatosis in males (about 40% micro-vacuolar and 60% of macro-vacuolar liver steatosis, Figure 1B) [30,31]. The lowest accumulation of fat in the liver of females after HFD was correlated with a better improvement of liver steatosis after Ole treatment (about 40% micro-vacuolar and 20% of macro-vacuolar liver steatosis in female vs. about 70% micro-vacuolar and 30% of macro-vacuolar liver steatosis Figure 1A,B). Biochemical parameters are reported in Table S1.

### 2.2. Gender Specific Activation of Akt/mTOR Pathway by Ole in ND and HFD Mice

First of all, we evaluated the expression of Akt and mTOR transcripts and functional proteins. 

**Figure 2:**
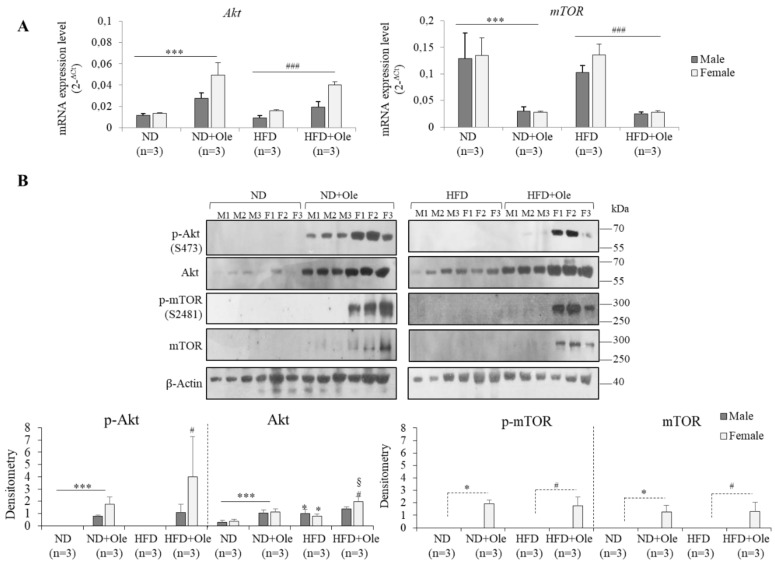
Gender-specific Akt/mTOR pathway in ND and HFD mice before and after Ole treatment. (**A**) *Akt* and *mTOR* mRNA expression level evaluated by Q-PCR in ND, ND + Ole, HFD and HFD + Ole male and female mice. Gene expression values are expressed as 2^−Δ*C*t^ values. The results are reported as mean ± SD. (**B**) Western blot analysis and relative densitometry of total and phosphorylated (p-Akt (S473), p-mTOR (S2481)) proteins in ND and HFD male (M) and female (F) mice, treated or not with Ole. Values are expressed as fold mean ± SD. * *p* < 0.05, *** *p* < 0.001 vs. ND; ^#^ *p* < 0.05, ^###^ *p* < 0.001 vs. HFD; ^§^ *p* < 0.05 vs. ND + Ole.

Surprisingly, in ND and HFD 16 week-mice, treated with Ole, the expression of total Akt protein was increased, and its activity was higher in females compared to males. After Ole treatment, only ND and HFD female mice, but not male mice, activated p-mTOR, showing a post-transcriptional regulation of this protein (Figure 2A,B).

The lack of Akt/mTOR pathway activation in 16-week-HFD mice could be due to the early stage of metabolic related damage. Thus, to support our hypothesis, the same mouse model was fed a HFD for 12 months, displaying, in accordance with the literature, a significant activation of Akt/mTOR pathway, either at transcriptional or at post-transcriptional levels (Figure S1). 

### 2.3. Activation of AMPK/ULK1 Pathway by Ole in ND and HFD Mice

AMPK is a metabolic stress-sensing enzyme involved in maintaining cellular energy homeostasis and interacts with phosphorylates, and activates the ULK1 protein kinase, a main initiator of the autophagic process. Thus, to understand if the mTOR sex-specific activation was involved in impairing liver autophagy we decided to look at the effects on AMPK/ULK1 intracellular pathway related to Ole treatment. 

**Figure 3:**
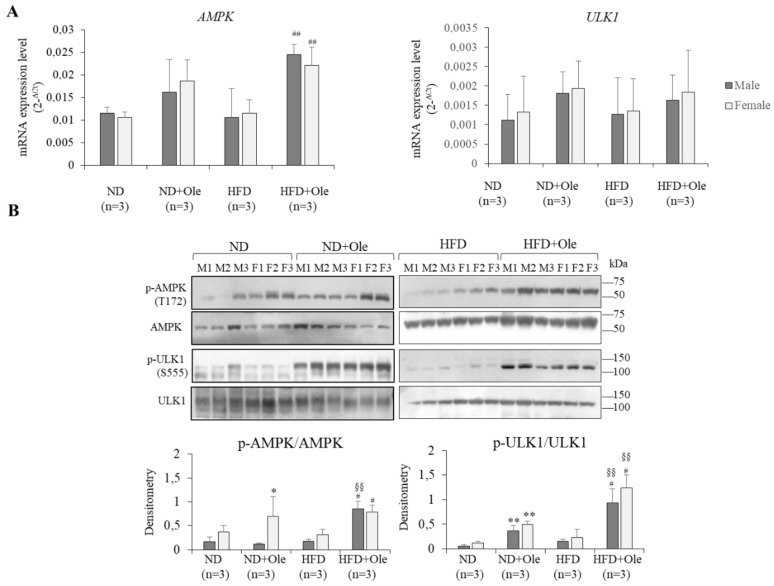
Activation of AMPK/ULK1 pathway by Ole in ND and HFD mice (**A**) *AMPK* and *ULK1* expression, evaluated by Q-PCR, in ND and HFD in male (M) and female (F) mice in presence or not of Ole. Gene expression values are expressed as 2^−Δ*C*t^ values. The results are reported as mean ± SD. (**B**) Western blot analysis and relative densitometry of pAMPK/AMPK and pULK1/ULK1 protein expression in ND and HFD in M and F mice in presence or not of Ole. Values are expressed as fold mean ± SD. * *p* < 0.05, ** *p* < 0.01 vs. ND; ^#^ *p* < 0.05, ^##^ *p* < 0.01 vs. HFD; ^§§^ *p* < 0.01 vs. ND + Ole.

Ole was able to initiate the autophagic process in ND and HFD 16 week-mice, regardless of the sex, by the increase of ULK1 phosphorylation at Ser555 (Figure 3A,B).

### 2.4. Ole is Able to Induce Early Autophagic Machinery

Afterwards, we examined the induction of autophagosome by Ole in ND and HFD mice. Q-PCR and Western blot analyses were performed on Beclin-1, LC3B and p62/Sqstm1.

**Figure 4:**
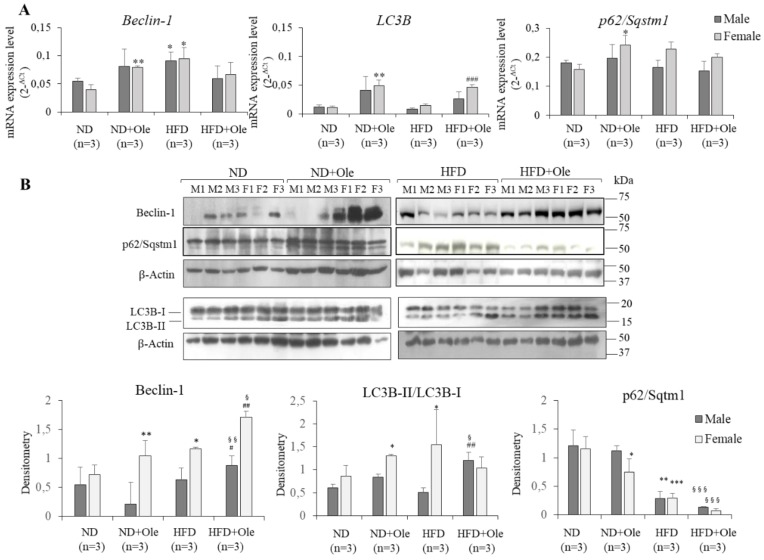
Ole induces early autophagic machinery (**A**) *Beclin-1*, *LC3B* and *p62/Sqstm1* expression, evaluated by Q-PCR, in ND and HFD in male (M) and female (F) mice in presence or not of Ole. Gene expression values are expressed as 2^−Δ*C*t^ values. The results are reported as mean ± SD. (**B**) Western blot analysis and relative densitometry of Beclin-1, LC3B-II/I and p62/Sqstm1 protein expression in ND and HFD in M and F mice in presence or not of Ole. Values are expressed as fold mean ± SD. * *p* < 0.05, ** *p* < 0.01, *** *p* < 0.001 vs. ND; ^#^ *p* < 0.05, ^##^ *p* < 0.01, ^###^ *p* < 0.001 vs. HFD; ^§^ *p* < 0.05, ^§§^ *p* < 0.01, ^§§§^ *p* < 0.001 vs. ND + Ole.

As shown in Figure 4, only in ND + Ole female mice, was Ole able to induce a significant increase of Beclin-1 and LC3B at transcriptional and post-transcriptional levels (Figure 4A,B). Accordingly, p62/Sqstm1 protein levels were down-regulated only in ND + Ole female mice (Figure 4B).

Interestingly, Ole treatment in 16-week-HFD mice was able to induce a significant up-regulation of Beclin-1 protein levels in both sexes (Figure 4B). In keeping with the increase of Beclin-1 protein, HFD + Ole mice displayed a significant up-regulation of LC3B-II/I protein level compared to ND + Ole mice (Figure 4B). Finally, a significant down-regulation of p62/Sqstm1 by Ole was observed (Figure 4A,B). 

### 2.5. Ole Does Not Affect the Expression of Caspase 3 and Bcl-2 Apoptotic Proteins in HFD Mice

We asked ourselves if the Akt protein activation, observed in female and male HFD-fed mice (see Figure 2), could be associated with the activation of apoptotic processes. To this aim, we looked at the *Caspase 3* and *Bcl2* transcriptional and post-transcriptional regulation.

**Figure 5:**
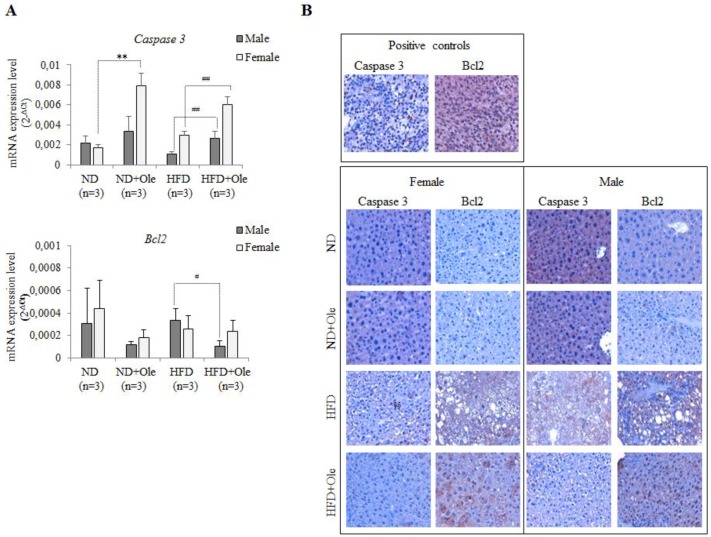
*Caspase 3* and *Bcl2* transcriptional expression. (**A**) *Caspase 3* (top) and *Bcl2* (bottom) expression, evaluated by Q-PCR, in ND and HFD mice, treated or not with Ole. Gene expression values are expressed as 2^−Δ*C*t^ values. The results are reported as mean ± SD. ** *p* < 0.01 vs. ND; ^#^ *p* < 0.05, ^##^ *p* < 0.01 vs. HFD. (**B**) Caspase 3 and Bcl2 expression in ND and HFD mice in presence or absence of Ole treatment. Immunohistochemistry in liver sections from representative female (left) and male (right) mice fed ND or HFD with or without Ole treatment. Original magnification × 400.

Interestingly, 16 weeks of HFD intake was not able to increase *Caspase 3* and *Bcl2* expression, whereas Ole treatment induced a significant up-regulation of *Caspase 3* mRNA expression level in ND + Ole female mice and in both sexes of HFD mice (Figure 5A).

Here, we would like to highlight that the increase of *Caspase3* in ND + Ole and in HFD + Ole mice follows the behaviour already appreciated for Beclin 1 after Ole administration.

*Bcl2* mRNA expression level was significantly decreased only in HFD + Ole male mice compared to the HFD group (Figure 5A).

Caspase 3 protein expression, investigated in liver tissues by immunohistochemistry (IHC) (Figure 5B), did not display any significant difference, whereas a weak increase of Bcl2 expression was noticed after Ole treatment (Figure 5B). However, the increase of Bcl2 protein expression did not reach statistical significance.

## 3. Discussion

Even if the knowledge on the autophagy process has been tremendously expanded over the past decades, the complex mechanisms involved are still far from understood. Moreover, the different sex-related behavior in inducing this safeguard cellular mechanism makes it even more difficult to understand the pathophysiological grounds [32]. Indeed, even if the effectiveness of controlling autophagy of several molecules, including phenol compounds [33] such as Ole, has been increasingly indicated, the bioavailability and metabolism of these compounds are variable and often dependent on a number of factors, including gender [26]. Thus, up to date, there are no available commercial drugs, able to activate or inhibit autophagy, approved by the US Food and Drug Administration (FDA) or equivalent regulatory agency [34].

Given these assumptions, we asked ourselves if the positive effects of Ole treatment, in the presence of liver steatosis [25], could be associated with the activation or inhibition of autophagy and if the biological effects were differently modulated in both sexes.

Ole treatment, in the presence of HFD intake, is able to induce autophagy response through the activation of AMPK/ULK1 pathway in both sexes.

Accordingly, it has been demonstrated that AMP-activated protein kinase (AMPK) induces autophagy by the phosphorylation of Atg1/ Unc-51-like-kinase1 (ULK1) at Ser555 [20]. On the other hand, recent studies have shown that insulin resistance can be regulated by Akt/mTOR pathway activation through a negative-feedback loop, but the mechanisms regulating this signaling through cellular energy are not as well defined as those for growth factors and nutrients [35].

Effectively, we highlighted that a mouse model fed HFD for 12 months exhibited a significant activation of the Akt/mTOR pathway (Figure S1) and was not more able to activate the autophagy proteins, such as Beclin-1 and LC3B. The results shown in Figure 2A,B indicate that the high calorie intake, at least at this initial phase of liver steatosis (16 weeks), as reported by Kimball et al. [36], induces an increased expression of Akt in both sexes not followed by mTOR activation. However, this phenomenon does not impair the ability of Ole to induce autophagy through the activation of AMPK/ULK1 intracellular pathway (Figure 3A,B).

In our opinion, all these results lend credence to a possible use of Ole in NAFLD patients, in keeping with previous data reported by Lim et al. that demonstrated the induction of autophagic process by an autophagy small-molecule enhancer (MSL) in a mTORC1-independent manner [37].

Finally, regarding the observed transcriptional activation of *Caspase 3* (Figure 5), several studies [11,38,39] demonstrated that Caspases-1, -3, and -7 seem to have a key role, not only in regulating apoptosis, but also the autophagic activity. Accordingly, it has been reported that autophagy dependent cell death, defined as a form of “non-protective autophagy”, seems to be slowed down by some pharmacological stimuli [40]. 

Concerning our study, we hope that it may start to shed light on the complex and still unclear autophagic biological world related to HFD in the presence or absence of drugs (such as Ole) demonstrating the ability to activate the autophagic process.

## 4. Materials and Methods

### 4.1. Mice Experimental Protocol

C57BL/6J mice, purchased from Charles River Laboratories International, Inc. (Wilmington, MA, USA) were housed in wire mesh cages maintained at controlled (21 ± 1 °C) temperature room with a 12 h light-dark cycle. Mice had ad libitum access to food and water.

After 1 week of acclimation, 24 mice were randomly divided into 4 groups (6 mice for each group, 3 male and 3 female) and fed with one of the following types of diet: 16 weeks of normal diet (TD.2018, Harlan; ND group), 8 weeks of normal diet + 8 weeks of normal diet and 3% of Ole dissolved in drinking water and daily administered by oral gavage (ND + Ole group), 16 weeks of high fat diet (TD.88137, Harlan; HFD group), 8 weeks of high fat diet + 8 weeks of high fat diet and 3% of Ole dissolved in drinking water and daily administered by oral gavage (HFD + Ole group). After 16 weeks, mice were sacrificed and each organ was dissected and samples were formalin-fixed for immune histological analysis or immediately frozen and stored at −80 °C until use for subsequent analysis.

All animal protocols were in accordance with the Guide for the Care and Use of Laboratory Animals and approved by the Institutional Animal Care and Use Committee at the University of Florence, Italy (178/2013 B, on 16 July 2013).

### 4.2. Histological Analysis

Specimens were formalin-fixed, paraffin-embedded and sectioned in order to assess the histological features by hematoxylin and eosin (H&E) staining analysis, using a standard protocol.

### 4.3. Immunohistochemistry for Caspase 3 and Bcl2

Formalin-fixed, paraffin-embedded sections (4 μm thick) were mounted on positively charged glass slides. The slides were cooled and endogenous peroxidase was blocked with peroxidase block buffer (citric acid 0.04 M, Na_2_HPO_4_·2H_2_O 0.12 M, NaN_3_ 0.03 M and H_2_O_2_ at 1.5% *v*/*v*) for 10 min at room temperature. Then, the sections were incubated for 1 h at room temperature with rabbit polyclonal antibody anti-Caspase 3 (Active) (1:20 dilution, 3015 Biovision, Milpitas, CA, USA) or with rabbit polyclonal antibody anti-Bcl2 (1:100 dilution, C21, sc-783, Santa Cruz Biotechnology, Dallas, TX, USA).

The primary antibodies were visualized using the avidin-biotin-peroxidase complex method (UltraTek HRP Anti-polyvalent, ScyTek, Logan, UT, USA) according to the instruction manual. 3,3′ diaminobenzidine was used as the enzyme substrate to observe the specific antibody localization, and Mayer hematoxylin was used as a nuclear counterstain. Negative controls were tissue sections stained in the absence of the primary antibody. Positive controls were A20 lymphoma cells injected subcutaneously in BALB/c mice. All samples were stained more than once and the results were highly reproducible. To assess differences in staining intensity, an immunoreactivity scoring system was applied. Intensity of staining was classified by both the percentages of the cells stained and the intensity of the staining [41]. In this way, the final scores of 0 to 3 were obtained (0, negative; 1, weak; 2, moderate; 3, strong.

### 4.4. RNA Extraction and cDNA Synthesis

Total RNA was extracted from liver tissues using Trizol reagent (Invitrogen, Carlsbad, CA, USA), according to the manufacturer’s instructions. One μg of total RNA was reverse transcribed using the High-Capacity cDNA Reverse transcription Kit (Applied Biosystems, Foster City, CA, USA), according to the manufacturer’s procedures.

### 4.5. Real-Time Quantitative Polymerase Chain Reaction PCR (Q-PCR) Analysis

Q-PCR analysis was performed by 7500 Fast Real-Time PCR System (7500 Software v2.0.5, Applied Biosystems) using Power SYBR™ Green PCR Master Mix (ThermoFisher Scientific, Waltham, MA, USA). For each sample, *β-Actin* Ct values was used for normalization purposes. For each gene, relative expression levels were computed as the difference (2^−Δ*C*t^) between the target gene Ct and *β-Actin* Ct.

Primers were designed using the PrimerQuest software (IDT Integrated DNA Technologies, Coralville, IA, USA) and purchased from BIO-FAB research (Rome, Italy). The following primers were used: 5′ *β-Actin*, 5′-GGGTCAGAAGGACTCCTATG-3′, 3′ *β-Actin*, 5′-GTAACAATGCCATGTTCA3′; 5′ *Beclin-1*, 5′-CAGGAGGAAGCTCAGTACCA-3′, 3′ *Beclin-1*, 5′-CTCCACACTCTTGAGTTCGT-3′; 5′ *LC3B*, 5′-CCCAGTGATTATAGAGCGATACA-3′, 3′ *LC3B*, 5′-GCAAGCGCCGTCTGATTAT-3′; 5′ *p62/Sqstm1*, 5′-CCACCAGAAGATCCCAATGT-5′; 3′ *p62/Sqstm1*, 5′-TCTCTTCCCTCCATGTTCCA-3′; 5′ *Akt*, 5′-GTAGCCATTGTGAAGGAGGG-3′, 3′ *Akt*, 5′-GCCGTTCCTTGTAGCCAATA-3′; 5′ *mTOR*, 5′-TTCTGGGGTGTTGGAATACG-3′; 3′ *mTOR*, 5′-CCACTCATGCAGCTTCTCAT-3′; 5′ *AMPK*, 5′-TCAGCACTCCGACAGACTTT -3′, 3′ *AMPK*, 5′-ACAGTAATCCACGGCAGACA-3′; 5′ *ULK1*, 5′-AGCACACGGAAACCCTACAC-3′; 3′ *ULK1*, 5′-AGCTCGAATCTGGTCAATGG-3′; 5′ *Caspase 3*, 5′-AGAGCACTGGAATGTCATCTC-3′, 3′ *Caspase 3*, 5′-CTTGGTATTTCAGGCCCATGA-3′; 5′ *Bcl2*, 5′-TTCAGGGATGGGGTGAACTG-3′, 3′ *Bcl2*, 5′-ATCCACAGGGCGATGTTGT-3′.

### 4.6. Western Blot Analysis

Total protein extraction was performed by homogenizing cells in Ripa lysis buffer containing 1X protease and phosphatase inhibitors cocktail (ThermoFisher Scientific, Waltham, MA, USA). The homogenates, after 30 min of incubation on ice, were then centrifuged at 13,000 rpm for 30 min at 4 °C. Protein concentrations were determined using the Bradford Protein Assay (Bio-Rad, Hercules, CA, USA). Lysates obtained from liver tissue were analyzed in denaturing condition through SDS-PAGE and transferred onto nitrocellulose membranes (Amersham Bioscience, Little Chalfont, UK). Membranes were incubated with primary antibodies followed by horseradish peroxidase-conjugate secondary antibody (Jackson Laboratories, Ann Arbor, MI, USA) and visualized with ECL (Western nova 2.0, Cyanagen, Italy). Densitometric analysis of immunoblots was performed by ImageJ64 image processing software for electrophoresis gel analysis.

Primary antibodies, diluted according to the manufacturer’s instruction, were as follows: β-Actin (sc-47778, C4) and Beclin-1 (sc-48341, E-8) purchased from Santa Cruz Biotechnology Inc. (Dallas, TX, USA), p-mTOR (Ser2481) (2974) and p-Akt (Ser 473) (9271) p-AMPK T172 (40H9), p-ULK1 (Ser555) (D1H4) and ULK1 (D8H5) from Cell Signaling (Danvers, MA, USA), LC3B (ab51520) and AMPK (ab3759) from Abcam (Cambridge, UK), and p62/Sqstm1 (PB9444) from Boster (Pleasanton, CA, USA). 

### 4.7. Statistical Analysis

Data were analyzed according to their distribution using different tools, i.e., parametric and non-parametric tests. To give major strength to our data we chose to express results as mean ± standard deviation (SD). The two-tailed Mann-Whitney test was applied to compare animal groups. Statistical significance was assessed by *p*-value thresholds: * *p* < 0.05; ** *p* < 0.01; *** *p* < 0.001. All statistical analyses were performed with Prism software version 6 (GraphPad Software, San Diego, CA, USA).

## Supplementary Materials

Supplementary materials can be found at http://www.mdpi.com/1422-0067/19/12/3948/s1.

## Abbreviations

ND	Normal diet
HFD	High-fat diet
Ole	Oleuropein
NAFLD	Non-alcoholic fatty liver disease
