# Supplementary material for: Oleuropein Induces AMPK-Dependent Autophagy in NAFLD Mice, Regardless of the Gender
Source: Int J Mol Sci. 2018 Dec 8;19(12):3948. doi: 10.3390/ijms19123948 (PMC6321282; doi:10.3390/ijms19123948)
Supplement: Supplementary file 1 [file ijms-19-03948-s001.zip › ijms-364448 supplementary/Table S1.pdf]

**Biochemical parameters of ND, ND+Ole, HFD and HFD+Ole Male mice**

| Parameters | ND | ND+Ole | HFD | HFD+Ole | *P* |
|---|---|---|---|---|---|
| Body Weight (gr) | 39.91 ±0.94 | 37.05 ±0.71 | 55.12 ±3.71 | 50.05 ±1.63 | 0,0006 |
| Liver Weight (gr) | 2.02 ±0.06 | 1.37 ±0.09 | 3.47 ±0.39 | 2.24 ±0.117 | 0,001 |
| Heart Weight (gr) | 0.19 ±0.01 | 0.16 ±0.01 | 0.286 ±0.015 | 0.19 ±0.014 | 0,05 |
| ALT (U/L) | 56.29 ±3.84 | 52.32 ±3.48 | 115.7 ±29.34 | 78.78 ±8.64 | 0,0004 |
| AST (U/L) | 226.89 ±58.90 | 264.16 ±52.01 | 268.84 ±30.86 | 263.77 ±40.95 | 0,005 |
| CHOL (mg/dL) | 155.13 ±10.95 | 148.91 ±9.75 | 192.79 ±23.74 | 160.16 ±12.93 | 0,001 |
| HDL (mg/dL) | 92.30 ±4.75 | 89.7 ±4.46 | 130.13 ±8.372 | 104 ±3.11 | 0,001 |
| LDL (mg/dL) | 9.36 ±0.84 | 9.07 ±0.88 | 14.43 ±2.18 | 12.48 ±1.3 | 0,014 |
| TRIG (mg/dL) | 113.36 ±8.77 | 108.42 ±8.28 | 130.91 ±9.68 | 115.96 ±7.95 | 0,001 |

**Biochemical parameters of ND, ND+Ole, HFD and HFD+Ole Female mice**

| Parameters | ND | ND+Ole | HFD | HFD+Ole | *P* |
|---|---|---|---|---|---|
| Body Weight (gr) | 36.53 ±1.352 | 30.16 ±1.31 | 47.45±2.04 | 44.2 ±2.28 | <0.0001 |
| Liver Weight (gr) | 1.96 ±0.13 | 1.72 ±0.17 | 2.96 ±0.23 | 1.92 ±0.14 | 0,0071 |
| Heart Weight (gr) | 0.16 ±0.003 | 0.15±0.0078 | 0.24 ±0.0026 | 0.15 ±0.0065 | 0,0066 |
| ALT (U/L) | 52.26 ±24.15 | 50.05 ±16.64 | 76.05 ±27.92 | 56.16 ±4.53 | 0,0004 |
| AST (U/L) | 261.01 ±62.55 | 198.83 ±58.52 | 265.62 ±25.25 | 261.04 ±75.62 | 0,005 |
| CHOL (mg/dL) | 149.5 ±10.14 | 145.99 ±9.36 | 238.91 ±12.96 | 174.33 ±13.55 | 0,0001 |
| HDL (mg/dL) | 82.16 ±4.86 | 90.40 ±4.34 | 126.52 ±4.14 | 94.38 ±5.13 | 0,0001 |
| LDL (mg/dL) | 10.4 ±1.41 | 10.01 ±1.35 | 13.39 ±2.054 | 11.83 ±1.10 | 0,001 |
| TRIG (mg/dL) | 72.40 ±6.70 | 84.6 ±6.78 | 117.78 ±11.20 | 102.18 ±13.36 | 0,0001 |

**Table S1.** Biochemical parameters of ND, ND+Ole, HFD and HFD+Ole Male (Top) and Female (Bottom) mice. Abbreviations: ALT, alanine aminotransferase; AST, aspartate aminotransferase; CHOL, Total cholesterol; HDL, High-Density Lipoprotein; LDL, Low-density lipoprotein; TRIG, triglycerides. Values are expressed as fold mean ±SD (*P< 0.05; **P< 0.01; ***P< 0.001).
