# Supplementary figures and images for: Oleuropein Induces AMPK-Dependent Autophagy in NAFLD Mice, Regardless of the Gender
Source: Int J Mol Sci. 2018 Dec 8;19(12):3948. doi: 10.3390/ijms19123948 (PMC6321282; doi:10.3390/ijms19123948)

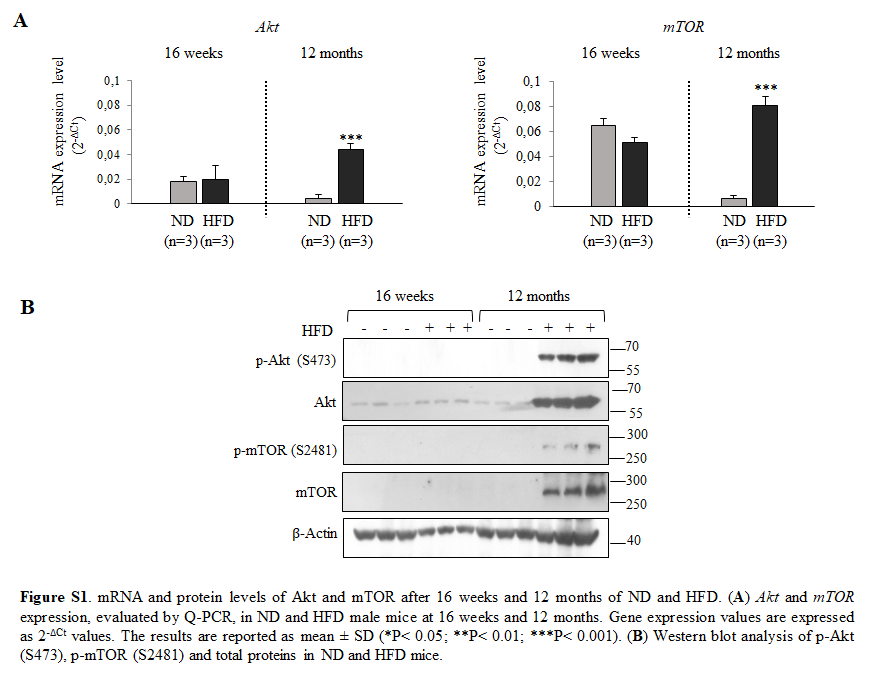

Supplement: Supplementary file 1 [file ijms-19-03948-s001.zip › ijms-364448 supplementary/Figure S1.tif]
